# A159 MALIGNANT PERITONEAL MESOTHELIOMA: A CASE OF MISTAKEN IDENTITY

**DOI:** 10.1093/jcag/gwad061.159

**Published:** 2024-02-14

**Authors:** A A Seeraj, A Cheung

**Affiliations:** Adult Gastroenterology, University of Ottawa Faculty of Medicine, Ottawa, ON, Canada; Adult Gastroenterology, University of Ottawa Faculty of Medicine, Ottawa, ON, Canada

## Abstract

**Background:**

In the Western hemisphere, cirrhosis is the most common cause of ascites. One of the least common causes is malignant peritoneal mesothelioma (MPM), which occurs in one in one million cases. MPM can be a diagnostic challenge due to its rarity and features that mimic other causes of ascites.

**Aims:**

To describe a complex case of MPM, highlighting the diagnostic dilemma stemming from the subtleties of presentation, confounders in ascites diagnostic criteria, and indeterminate testing.

**Methods:**

We performed a detailed retrospective chart review of a patient who presented with ascites. He was initially given the diagnosis of decompensated cirrhosis and eventually was diagnosed with MPM. He provided his consent for this case report.

**Results:**

A 37-year-old male presented with progressive ascites and peripheral edema. He had no known exposure to asbestos and consumed 6 standard drinks a day for 2 years; with a prior history of 10 standard drinks a week for over 3 years. His physical examination was unremarkable for cardiac, renal or liver disease. His transthoracic echocardiogram and urinalysis were normal. Abdominal ultrasound showed features of liver cirrhosis with large-volume ascites and a FIB-4 score of 0.34, excluding advanced fibrosis Laboratory investigations including liver tests were normal; with a platelet count of 568 x 10^9^/L. Viral, metabolic and autoimmune liver disease were excluded. A diagnostic paracentesis demonstrated a serum albumin-ascites gradient (SAAG) of 1.3 g/dL. Due to his history of alcohol misuse, imaging findings and high SAAG, he was diagnosed with alcohol-related cirrhosis.

His repeat abdominal ultrasound showed multiple liver nodules. Magnetic resonance imaging was done to investigate for hepatocellular carcinoma; revealing diffuse peritoneal carcinomatosis, cirrhosis and large-volume ascites. Investigations to identify the primary malignancy included a computed tomography chest, colonoscopy and EGD. His EGD was the only positive test; showing a 2cm submucosal gastric lesion with normal gastric mucosa pathology. An endoscopic ultrasound (EUS) with fine needle aspiration was then performed; the lesion appearance in keeping with a gastrointestinal stromal tumor (GIST). Pathology favored a diagnosis of poorly differentiated gastric carcinoma. Cytology was positive for malignancy, with the differential being mesothelioma or adenocarcinoma and a repeat SAAG was 1.0 g/dL. Given the discordances, an ultrasound-guided core biopsy was performed of the peritoneal lesions. Pathology revealed features of poorly differentiated epithelioid mesothelioma. A subsequent review of his gastric biopsies revealed similar cells in retrospect were in keeping with mesothelioma.

**Conclusions:**

The presentation of MPM is not easily distinguishable from other causes of ascites. There must be a high degree of suspicion for malignant ascites in the face of inconsistent clinical and diagnostic findings.

**Funding Agencies:**

None

